# Using the Tools we Have: Low-efficacy Vaccines and HIV

**DOI:** 10.1016/j.ebiom.2015.12.007

**Published:** 2015-12-12

**Authors:** Ryan Zurakowski

**Affiliations:** Department of Biomedical Engineering, University of Delaware, Newark, DE 19716, USA

**Keywords:** HIV vaccine, Mathematical modeling

Mathematical modeling has played an important role in the fight against HIV. Mathematical analysis of a protease inhibitor experiment enabled the development of the first suppressive multi-drug regimens for treating HIV (Ho et al., 1995). Mathematical model use in disease epidemiology dates back almost a century (Kermack and McKendrick, 1927). Mathematical modeling allows us to rigorously explore the implications of complex hypotheses. Predictive models are particularly useful in epidemiology, where multi-armed experiments in vaccination may be impractical or unethical (Scherer and McLean, 2002).

It is in this tradition that Dimitrov, Kublin, Ramsey and Corey present an analysis of potential vaccination strategies for HIV in *EBioMedicine* (Dimitrov et al., 2015) The search for an effective HIV vaccine has been long (Fauci and Marston, 2015), (Haynes, 2015). Nearly 30 years have passed since the first HIV vaccine trials, and we have only recently found the first vaccine candidate with any measurable protection against HIV infection in human subjects (Rerks-Ngarm et al., 2009), and the reported efficacy of 31% is too low for regulatory approval. It is likely that incremental advances based on this partial success will soon result in a vaccine with adequate protection, at least for one clade of the virus. As Dimitrov and colleagues point out, however, it is unlikely that a single vaccine will provide the same level of protective immunity to different HIV clades, which vary between geographic regions.

If this is the case, then a decision will need to be made whether to introduce a less effective vaccine, or to wait until a more effective vaccine becomes available. Dimitrov and colleagues present a mathematical model of HIV spread through populations, including ranges of possible population behaviors matched to data from HIV surveillance studies in San Francisco and South Africa. They compare three vaccine policies: a vaccination policy where a low efficacy vaccine is introduced immediately and maintained for 10 to 30 years, a vaccination policy where the low efficacy vaccine is introduced immediately and switched for a moderate efficacy vaccine when it becomes available 3–8 years later, and a policy where no vaccine is introduced until the moderate efficacy vaccine is available.

The strategies that vaccinated early and then switched are predicted to be the most effective overall, resulting in a median of 5% fewer total infections by year 30 when compared with the delayed strategies, a benefit which was remarkably consistent across different possible population behaviors. This represents billions of dollars in savings measuring antiretroviral therapy costs alone, not to mention the savings in morbidity and mortality.

These results are a strong argument for early introduction of even partially effective vaccines, but this is by no means a closed discussion. If a predictive model neglects significant effects, then reality will deviate from the model predictions. As an example, the authors considered the possibility that vaccinated patients may reduce their condom use compared with unvaccinated patients. The authors show that a 50% reduction in condom use among vaccinated patients erases the benefit of the immediate vaccination strategy, making delayed vaccination the preferred strategy. This could be moderated through a campaign aimed at preventing the reduction of condom use by vaccinated persons, but this is a non-trivial hurdle for implementation. The campaign would have to maximize vaccine coverage while simultaneously emphasizing the low protective benefit of the vaccine to maintain high condom use in the population. Furthermore, the sensitivity of the results to this one secondary effect emphasizes the need to carefully consider all possible secondary effects, to reduce the chances of missing a factor which could lead to failure in the field. More work is therefore necessary before deciding on a vaccine policy. Nevertheless, the authors have made a compelling case for the potential benefits of deploying low efficacy vaccines for HIV, an option which would never have been considered otherwise.
